# Defining the Structural Stability Field of Disordered Fluorite Oxides

**DOI:** 10.3389/fchem.2021.733718

**Published:** 2021-08-19

**Authors:** Eric C. O’Quinn, Devon L. Drey, Maik K. Lang

**Affiliations:** Department of Nuclear Engineering, University of Tennessee, Knoxville, TN, United States

**Keywords:** fluorite, structural stability, complex oxides, oxygen vacancy, defects, Goldschmidt, Pauling

## Abstract

Fluorite-structured oxides constitute an important class of materials for energy technologies. Despite their high level of structural symmetry and simplicity, these materials can accommodate atomic disorder without losing crystallinity, making them indispensable for uses in environments with high temperature, changing chemical compositions, or intense radiation fields. In this contribution, we present a set of simple rules that predict whether a compound may adopt a disordered fluorite structure. This approach is closely aligned with Pauling’s rules for ionic crystal structures and Goldschmidt’s rules for ionic substitution.

## Introduction

Materials that are isostructural with the mineral fluorite (CaF_2_) have been studied for nearly 100 years and were instrumental to Pauling (1927) and Goldschmidt (1926) in development of the first sets of atomic radii. The extraordinarily simple structure consists of a cubic array of anions, with cations filling every other cubic interstice (Navrotsky, 2010). An even simpler representation involves stacking of atomic layers in the sequence anions-cations-anions anions-cations-anions (Sickafus et al., 2005; Uberuaga and Sickafus, 2015); these layers are perfect triangular Ising nets (Wannier, 1950). While many metal cations form binary fluorite oxides (*e.g.,* CeO_2_), the flexibility of the fluorite structure permits the formation of ternary or even higher-entropy (*i.e.,* more than one cation) fluorite oxides and hyper- or hypo-stoichiometric (*i.e., M*O_2±*x*_) oxygen sublattices. Based on the atomic disorder involved, these compounds have been referred to as disordered fluorites (Norberg et al., 2012; O’Quinn et al., 2020), anion-deficient fluorites (Sickafus et al., 2007; Tang et al., 2007), or defect fluorites (De Los Reyes et al., 2013). Hereafter, we describe these as “disordered fluorites,” a general term encompassing fluorite structures in which structural disorder is observed on either the cation sublattice, the anion sublattice, or both. A classic example is the well-known disordered fluorite yttria-stabilized zirconia (Y_*x*_Zr_1-*x*_O_2-0.5*x*_, “cubic zirconia” or “YSZ”). In YSZ, despite the hypo-stoichiometric anion sublattice, the remaining oxygen readily forms a simple cubic framework in which the two metal cations, Y^3+^ and Zr^4+^, distribute themselves randomly (Götsch et al., 2016). Conversely, another well-known example is the nuclear fuel uranium dioxide (UO_2+*x*_ + fission products). In a nuclear reactor, the fission process leads to continuous incorporation of metal cations into the fluorite lattice, oxidation of the U^4+^ cations, and a hyper-stoichiometric anion sublattice. Notwithstanding the complex chemistry of both cases, their overall structure remains remarkably simple (Navrotsky, 2010). Disordered fluorite oxides exhibit a variety of useful physical properties such as high ion conduction (van Dijk et al., 1983), low thermal conductivity (Clarke and Phillpot, 2005), and excellent radiation tolerance (Sickafus et al., 2000) which permits their use as fuel cell electrolytes (Navrotsky, 2010), thermal barrier coatings (Xu et al., 2006), and nuclear fuels (Ewing et al., 2004). Despite the prevalence and importance of this material class, there has been limited efforts to predict the structural stability field of disordered fluorite oxides.

The structural stability field of fluorite-derived structures has been previously defined only with respect to other ordered fluorite-derivative superstructures (Minervini et al., 2000; Yamamura et al., 2003). For instance, the conditions that define the boundary between pyrochlore and fluorite oxides in *A*_2_ *B*_2_O_7_ compounds has been the source of much investigation (Isupov and Petrov, 1958; Heremans et al., 1995; Minervini et al., 2000; Sickafus et al., 2000; Pirzada et al., 2001; Cai et al., 2011; Mouta et al., 2013; Fuentes et al., 2018). The ordered pyrochlore structure can be imagined as a 2 × 2 × 2 supercell of the fluorite unit cell in which the stoichiometric oxygen vacancy is ordered at a specific location creating distinct coordination polyhedra for the two cations: an 8-fold cubic polyhedron for the larger A-cation and a smaller 6-fold octahedron for the B-cation. The commonly used rule to explain or predict the boundary of the compositionally-induced transition between disordered fluorite ordered and ordered pyrochlore is when the ionic radius ratio of the 8-coordinated A-site cation (conventionally Shannon, 1976) and the 6-coordinated B-site cation exceeds 1.46 (Subramanian et al., 1983; Minervini et al., 2000; Sickafus et al., 2000; Fuentes et al., 2018). Experimental and computational studies have shown that this ionic radius ratio is strongly correlated with the cation anti-site defect formation energy in *A*_2_ *B*_2_O_7_ (Minervini et al., 2000; Sickafus et al., 2000) with an increased propensity for a disordered cation sublattice and a disordered fluorite structure for lower defect energies. The ionic radius ratio is a simple way to predict if a disordered fluorite structure forms for a given *A*_2_ *B*_2_O_7_ composition. A similar set of guiding principles is lacking for other stoichiometries and classes of materials.

**FIGURE 1:**
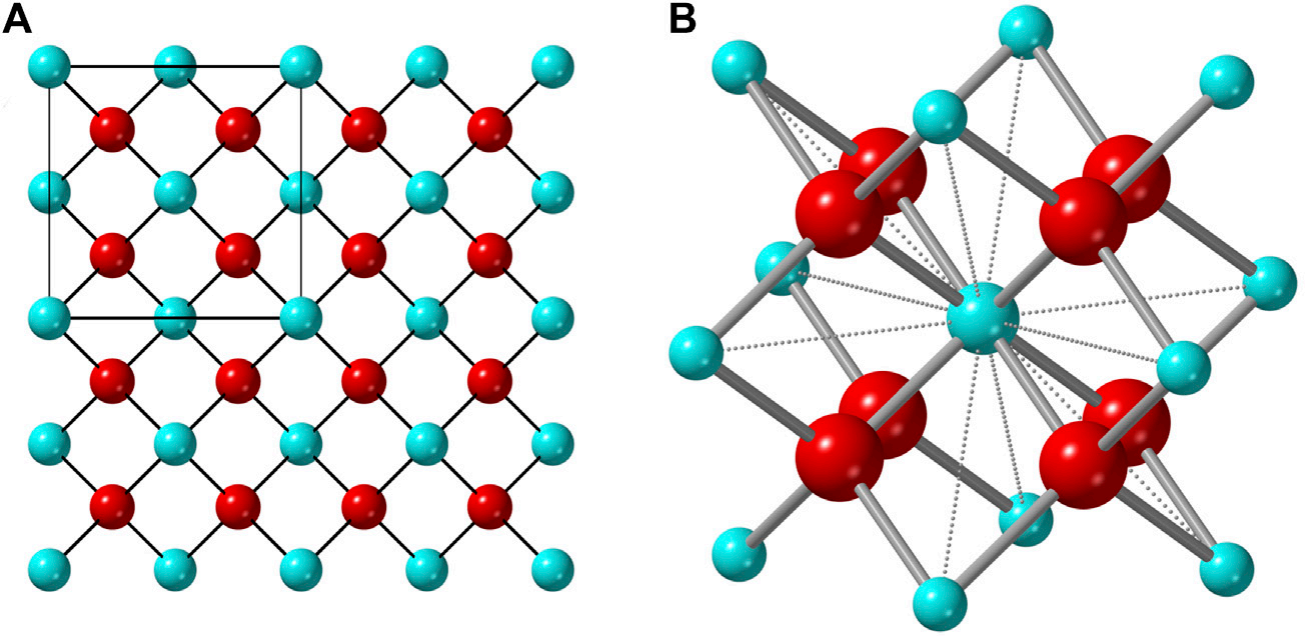
**(A)** Atomistic representation of the ideal fluorite structure with the unit cell denoted by the square in the upper left corner. **(B)** The cube-octahedron represented by a polyhedron formed by twelve vertices occupied by cations (blue) and the nearest eight anions (red) to the central cation (same for any other cation).

An ionic compound that exhibits a stable (or metastable) disordered structure must contain a geometric framework that permits mixing of the cation and/or anion sublattices. For the disordered fluorite structure, this is the interpenetrating face-centered-cubic cation array and simple cubic anion array (Figure 1); the repeat unit of the cation sublattice is twice that of the anion sublattice. Any cation in a fluorite structure is nearest neighbors with 12 other cations which form a cube-octahedral cage in which the eight nearest-neighbor anions reside. This geometry creates eight regular tetrahedra, emerging from the center cation, in which the anions reside at the circumcenter (*i.e.,* all cation-anion distances are equal). Only within the ideal fluorite structure, the anions occupy the center of these tetrahedron, and other structurally related phases (*e.g.,* pyrochlore and bixbyite) form if anions move away from their ideal positions. This process is highly correlated with the size of the cations that form the cube-octahedral cage. If some of the constitutional cations are too large (or small) compared with the cations forming the rest of the cube-octahedral cage, the enclosed anions will relax away from the center of the tetrahedron, distorting the ideal simple cubic anion array. The second characteristic of the disordered fluorite structure is the random mixing of cations on one site. The size of the two cation species must be balanced to allow for occupation of the same site but prevent both cation-anion and anion-anion repulsion, or “double repulsion.” This effect can be easily conceptualized in Figure 1: any cation-cation distance forms a shared edge of two anion tetrahedra. If the cations are both sufficiently large, then nearest-neighbor cation-anion repulsive forces will keep the nearest-neighbor anions from close contact. If the cations are too small, however, both cation-anion and anion-anion repulsive forces (double repulsion) prevent the disordered fluorite structure from being adopted. Pauling (1960) observed that the phase boundary for *MX*_2_ compounds between fluorite (cations in 8-fold coordination) and rutile (cations in 6-fold coordination) was explained if the ionic radii ratio of cations and anions is approximately $\sqrt{3}-1=0.732$, the lower geometric limit to prevent double repulsion.

We expand this approach here and show that simple geometrical constraints aid in the formulation of simple rules to define the structural stability field of disordered fluorite. 1) For compounds with more than one cation species, the size of any cation must not be too different from the average size of all other cations; this maintains the ideal simple cubic anion array within the cube-octahedral cage. This rule can be understood as an enforcement of Goldschmidt’s first rule (Goldschmidt, 1926) that free ionic mixing in an ionic crystal is limited to cases where the relative ion size is no larger than 15%. The potential for different cations to mix over the same site is also limited by Pauling’s third and fourth rule which states that small, high-valence cations tend not to share polyhedral edges, as is done in the fluorite structure. 2) The average size of the cations in a compound must be sufficiently large that an 8-fold coordination with the anion sublattice is preferred. 3) The average size of the cations in a compound must be sufficiently small to prevent ordering to higher coordination numbers. These last two rules are an application of Pauling’s first rule (Pauling, 1929) which prescribes different coordination polyhedra for cations based on their relative size to their surrounding anions. In this contribution we demonstrate how the simple rules (*I*–*III*) (known for nearly a century) can be used to predict the stable phase region of disordered fluorite for any given complex oxide based on their chemical composition and stoichiometry. This methodology provides an easy, accessible framework that is based on Pauling’s and Goldschmidt’s original rules to guidance the synthesis of novel, disordered fluorite-structured materials.

## Methodology

To define the structural stability field of disordered fluorite, we identified several parameters by which chemical compounds are uniquely characterized. Given the three rules proposed in the introduction, we focused on characterizing the relative sizes of the constituent ions to one another. We used the Pauling univalent radii (Pauling, 1929), which describe the relative sizes of the outermost electron shells of a given ion (Pauling, 1960). They are referred to as “univalent” because ions are treated as though they have maintained their electron distribution but also have Coulombic interaction as though they had a charge state of ±1. Thus, this radius represents a measure of the relative spatial extensions of ions and their characteristic repulsive forces; thus, Pauling originally used the univalent radii to evaluate the second component of his first rule (“the no-rattle rule”). The Pauling univalent radius was calculated for a range of ions (Table 1) using the relation (Eqs 13–8 in Ref. Pauling, 1960):

$$R_{z}=R_{1}z^{-2/\left(n-1\right)}$$ (1)

where *R*_*z*_ is the Pauling empirical crystal radius, *R*_*1*_ is the Pauling univalent radius, *z* is the charge state of the ion, and *n* is the Born exponent (Table 13-2 in Ref. Pauling, 1960).

**TABLE 1:** Summary of the different radii used in this study. The previously unreported Pauling univalent radii were calculated from Eqs 13–8 in Pauling (1960) using a value of 12 for the Born exponent (corresponding to an electron configuration), in accordance with Table 13-2 in Pauling (1960). The Pauling empirical crystal radii are from References Pauling (1929), Pauling (1960), Galasso (1970), and Rohrer (2001). Entries in bold represent previously unreported data and were calculated for this study.

Atomic number	Atom species	Charge state	Pauling empirical crystal radius (Å)	Pauling univalent radius (Å)
8	O	−2	1.40	1.76
22	Ti	+4	0.68	0.96
39	Y	+3	0.93	1.20
40	Zr	+4	0.80	1.09
41	Nb	+5	0.70	1.00
50	Sn	+4	0.71	0.96
51	Sb	+5	0.62	0.89
57	La	+3	1.15	**1.40**
58	Ce	+3	1.11	**1.36**
59	Pr	+3	1.09	**1.33**
60	Nd	+3	1.08	**1.32**
61	Pm	+3	1.06	**1.29**
62	Sm	+3	1.04	**1.27**
63	Eu	+3	1.03	**1.26**
64	Gd	+3	1.02	**1.25**
65	Tb	+3	1.00	**1.22**
66	Dy	+3	0.99	**1.21**
67	Ho	+3	0.97	**1.18**
68	Er	+3	0.96	**1.17**
69	Tm	+3	0.95	**1.16**
70	Yb	+3	0.94	**1.15**
71	Lu	+3	0.93	**1.14**
72	Hf	+4	0.81	**1.04**

Two parameters were then calculated for various chemical compounds; first, a parameter was defined to quantify the relative size of a given cation *to all other cations, themselves*; we did this by the following relation:

$$\begin{array}{l} \rho_{cation-cation}=\left(i\right)\;\frac{\langle r_{all\;cations}\rangle }{\langle r_{large\;cations}\rangle }\;if\;\mathrm{more}\;\mathrm{cations}\;\mathrm{are}\;\mathrm{larger}\;\mathrm{than}\;\mathrm{the}\;\mathrm{average}\\ or\\ \left(ii\right)\;\frac{<r_{small\;cations}>}{<r_{all\;cations}>}\;if\;\mathrm{more}\;\mathrm{cations}\;\mathrm{are}\;\mathrm{smaller}\;\mathrm{than}\;\mathrm{the}\;\mathrm{average}\; \end{array}$$ (2)

where the values in angled brackets (< >) are the average radius, which is calculated either for (*i*) all cations larger or (*ii*) all cations smaller than the average cation size. For the example of the disordered, anion-deficient fluorite Yb_3_NbO_7_ compound, case *(i*) applies as the majority of cations are larger (Yb) than the average cation size. Conversely, for the example of yttria-stabilized zirconia, case (*ii*) applies because the majority of cations are Zr with a smaller radius than the average cation size. For complex oxides with equal numbers of cations that are larger and smaller than the average cation size (*e.g.,* Ho_2_Zr_2_O_7_), case (*i)* was applied. A second parameter was defined, ρ_cation-anion_, to quantify the relative size of the cations *to the anions*:

$$\rho_{\mathrm{cation}-\mathrm{anion}}=\frac{\langle r_{\mathrm{all}\;\mathrm{cations}}\rangle }{\langle r_{\mathrm{all}\;\mathrm{anions}}\rangle }$$ (3)

This requires an average anion radius which must be determined considering any anion vacancies in the disordered fluorite structure. In an ideal fluorite structure (*i.e.,* MX_2_), the average anion radius coincides with the actual anion radius because all anion positions are occupied. This changes for anion-deficient fluorite structures because the average anion radius must account for the vacant positions on the anion sublattice. For example, the average anion radius for a disordered M_4_O_7_ fluorite structure is $\frac{7}{8}r_{O}$; conceptually this means that 7 out of 8 anions have a radius equal to the oxygen radius and the 8th anion has a radius equal to 0. It should be clarified that the radius of the vacant anion is not a “vacancy radius,” or radius of the resulting void space; instead, it is a “zero radius,” the radius of no anion at all. Thus, Eq. 3 may be interpreted as a generalization of Pauling’s first (“no rattle”) rule for more complex cases involving, for instance, anion vacancies.

A phase space was created with *ρ*_cation-cation_ (hereafter referred to as *ρ*_*c*_) on the abscissa and *ρ*_cation-anion_ (*ρ*_*a*_) on the ordinate to incorporate all possible disordered fluorite structures for different complex oxides and infer stability boundaries related to cationic and anionic radii. This procedure is illustrated for the example of the disordered, anion-deficient fluorite Y_2_Zr_2_O_7_ compound. First, the average size of the two cations is determined by:

$$\frac{2r_{Y}+2r_{Zr}}{4}=1.145$$

Then the parameter *ρ*_*c*_ is given by:

$$\frac{1.145Å}{r_{Y}}=0.954.$$

Finally, the parameter *ρ*_*a*_ is calculated as:

$$\frac{1.145Å}{r_{O}*\frac{7}{8}}=0.743,$$

with *r*_Y_ = 1.20 Å, *r*_Z_ = 1.09 Å, and *r*_O_ = 1.76 Å (Table 1).

## Results and Discussion

**FIGURE 2:**
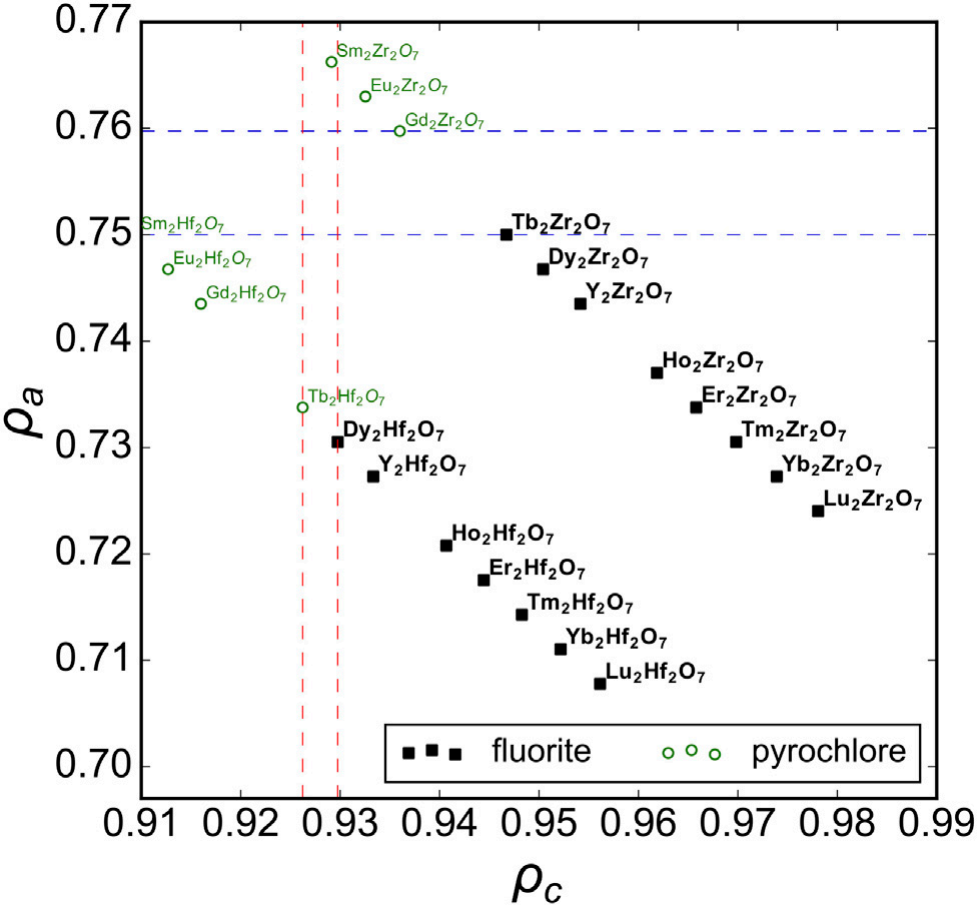
The phase space spanned by the relative size of cations to anions, ρ_a_, *versus* relative size of cations to one another, ρ_c_, for A_2_B_2_O_7_ oxides across a broad range of chemical compositions with experimental data compiled from Roth (1956), Klee and Weitz (1969), Subramanian et al. (1983), Minervini et al. (2000), Sickafus et al. (2000), Stanek and Grimes (2002), Ewing et al. (2004), Reynolds et al. (2013), and Drey et al. (2020). Compounds reported with a structure corresponding to disordered fluorite and ordered pyrochlore are represented by solid black squares and open green circles, respectively. The vertical dashed red lines represent the ρ_c_ boundaries based on ternary hafnates and the horizontal dashed blue lines the ρ_a_ boundaries based on ternary zirconates.

First, we consider the well-studied *A*_2_ *B*_2_O_7_ family of oxides as a model system to probe the structural stability field of disordered fluorite. Previous studies have established the phase boundary between ordered pyrochlore and disordered fluorite across a range of chemical compositions (Subramanian et al., 1983). We used Pauling’s univalent radii (Figure 2) to re-examine these well-studied ternary oxides and to create a phase space based on the sizes of the cations relative to *one another* (*ρ*_*c*_ – abscissa) and with respect to the oxygen anions (*ρ*_*a*_ – ordinate). Experimental data show that ternary hafnate oxides (*A*_2_Hf_2_O_7_) exhibit a disordered fluorite structure for *A* = Dy-Lu and Y (Klee and Weitz, 1969; Stanek and Grimes, 2002; Ewing et al., 2004), while ordered pyrochlore forms for A = La-Tb. In our phase space, this corresponds to a phase boundary of *ρ*_*c*_ = 0.928(2) (*i.e.,* 0.928 ± 0.002) which is the average value obtained by the two *ρ*_*c*_ values of the neighboring disordered fluorite and ordered pyrochlore compounds (Figure 2, red vertical lines). When the two cations become more different in size than Dy and Hf, the same coordination polyhedra simply cannot accommodate both cations and maintain the configuration of disordered fluorite, and an ordered pyrochlore structure forms. For the ternary zirconate oxides (*A*_2_Zr_2_O_7_), the disordered fluorite structure is adopted for *A* = Tb-Lu, and Y (Roth, 1956; Klee and Weitz, 1969; Subramanian et al., 1983; Ewing et al., 2004; Reynolds et al., 2013). In Figure 2, the boundary between the ordered pyrochlore Gd_2_Zr_2_O_7_ and the disordered fluorite Tb_2_Zr_2_O_7_ corresponds to a *ρ*_*c*_ = 0.950(4) or an A-site cation radius ∼13% larger than that of the B-site cation. However, another way to discriminate the two compositions is based on their *ρ*_*a*_ values (Figure 2, blue horizontal lines). The phase boundary in this case is when *ρ*_*a*_ = 0.755(5), which means that the average cation size is ∼75.5% the average anion size. If this value is exceeded, the cation sublattice will prefer a different coordination scheme that provides the larger cation of the two with more anion neighbors (*i.e.,* pyrochlore). In this way, the phase space of disordered fluorite is defined with boundaries to ordered pyrochlores based on the size of the cations with respect to the oxygen in the ternary zirconates and with the relative size among the two cations for ternary hafnates.

**FIGURE 3:**
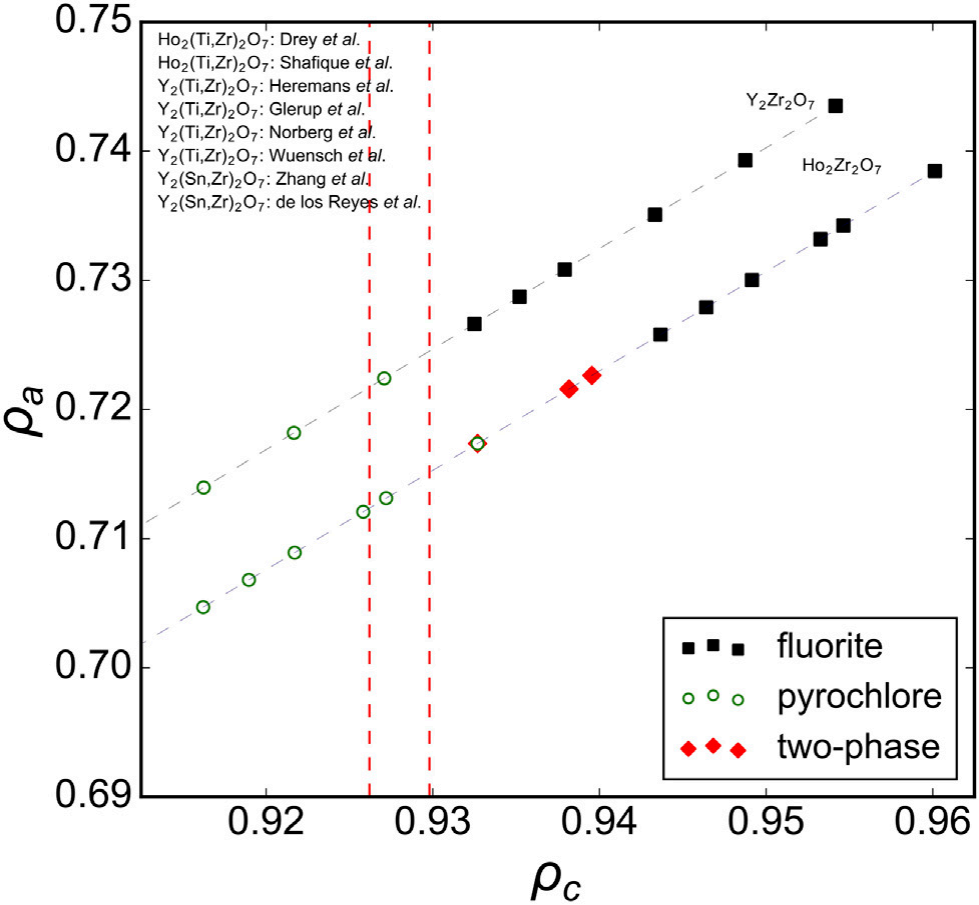
Ternary A_2_B_2_O_7_ solid solution series in the ρ_a_, ρ_c_ phase space with ρ_c_ boundaries (red dashed vertical lines) established from A_2_Hf_2_O_7_ compositions (Figure 2). Experimental data taken from Heremans et al. (1995), Kennedy et al. (1997), Wuensch et al. (2000), Glerup et al. (2001), Norberg et al. (2012), De Los Reyes et al. (2013), Zhang et al. (2013), Shafique et al. (2016), Tracy et al. (2016), and Drey et al. (2020) with reported structures that correspond to disordered fluorite (solid black squares), ordered pyrochlore (open green circles), and a mix of both (red diamonds). The dashed gray lines connect the data points and represent the trend of the solid-solution series within this phase space.

Analyzing previous studies on solid-solution series of ternary *A*_2_ *B*_2_O_7_ oxides covering the full range between ordered and disordered structures yields more insight to the compositional phase boundaries of disordered fluorite. For instance, Figure 3 shows several solid solutions plotted in the phase space spanned by *ρ*_*a*_ and *ρ*_*c*_ with the *ρ*_*c*_ boundary lines overlaid from the ternary hafnates (Figure 2). A recent study by Drey et al. (2020) examined a solid solution between the ordered pyrochlore Ho_2_Ti_2_O_7_ and disordered fluorite Ho_2_Zr_2_O_7_; Ti-rich compositions adopted the pyrochlore phase, Zr-rich compositions adopted the fluorite phase, and intermediate compositions were shown to have a combination of both. The compositional boundary between ordered pyrochlore and disordered fluorite lies in a narrow *ρ*_*c*_-range. Other studies have investigated compositionally similar series (*e.g.,* Ho_2_Ti_2_O_7_-Ho_2_Zr_2_O_7_ Shafique et al., 2016 and Y_2_Ti_2_O_7_-Y_2_Zr_2_O_7_ Heremans et al., 1995; Wuensch et al., 2000; Glerup et al., 2001; Norberg et al., 2012); the boundary between pyrochlore and fluorite in these series exists within the same narrow *ρ*_*c*_-range. Some studies included ternary stannates (Y_2_Sn_2_O_7_-Y_2_Zr_2_O_7_) which have a more covalent bond character than zirconates and titanates (De Los Reyes et al., 2013; Zhang et al., 2013). It was demonstrated that the phase boundary between disordered fluorite and ordered pyrochlore is very similar to the Y_2_Ti_2_O_7_-Y_2_Zr_2_O_7_ and Ho_2_Ti_2_O_7_-Ho_2_Zr_2_O_7_ series and occurs at the same Zr-content levels. While Sn^4+^ has a larger ionic radius than Ti^4+^ (0.690 Å *versus* 0.605 Å), Sn cations have the same univalent radius as Ti cations (0.96 Å). This shows again that the univalent radius is a very useful parameter in determining the structural stability of disordered fluorite.

**FIGURE 4:**
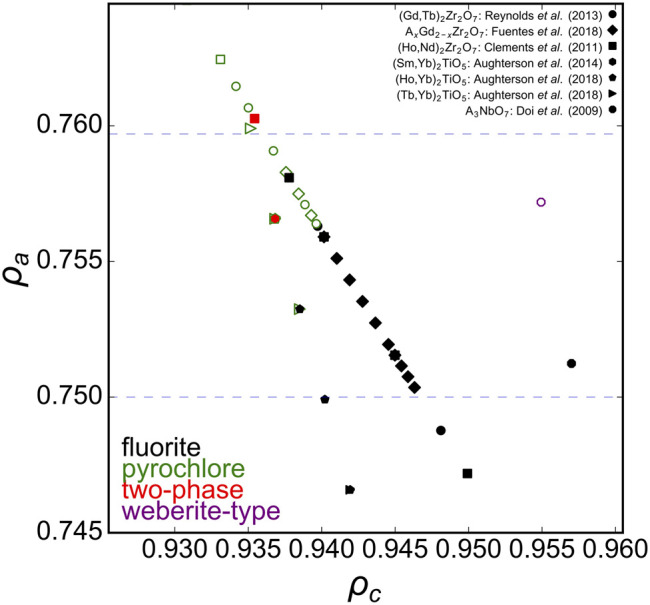
Ternary A_2_B_2_O_7_ solid solution series in the ρ_a_, ρ_c_ phase space with ρ_a_ boundaries (blue dashed horizontal lines) established from A_2_Zr_2_O_7_ compositions (Figure 2). Experimental data points represented by diamonds taken from Fuentes et al. (2018), circles from Reynolds et al. (2013), squares from Clements et al. (2011), hexagons from Aughterson et al. (2014), pentagons from Aughterson et al. (2018a), triangles from Aughterson et al. (2018b), and octagons from Doi et al. (2009). Solid black squares represent disordered fluorite, open green squares pyrochlore, and solid red squares a mix of both.

As mentioned above, the phase boundary of disorder fluorite with respect to the univalent radius *ρ*_*a*_ was determined with different *A*_2_Zr_2_O_7_ zirconate oxides (Figure 2 blue, dashed horizontal lines). Solid solutions involving substitution of the A-cation in A_2_Zr_2_O_7_ with many intermediate compositions are useful to further probe this phase boundary (Figure 4). Reynolds et al. (2013) studied the long-range structure of series members between the ordered pyrochlore Gd_2_Zr_2_O_7_ and disordered fluorite Tb_2_Zr_2_O_7_. Fuentes et al. (2018) probed similar zirconate oxides (*A*_x_Gd_2-x_Zr_2_O_7_) and based on the experimental data of both studies, the phase boundary for the stability field of disordered fluorite occurs at *ρ*_*a*_ ∼ 0.756. This agrees very well with work by Clements et al. (2011) who investigated a solid solution of ordered Nd_2_Zr_2_O_7_ and disordered Ho_2_Zr_2_O_7_ and reported a critical transformation from ordered pyrochlore to disordered fluorite that corresponds, in our phase space calculated with univalent radii, to 0.757 < *ρ*_*a*_ < 0.760. Intriguingly, this value represents also the phase boundary of disordered fluorites that form in other families of oxides such as the ternary niobates with the general formula *A*_3_NbO_7_, for which the disordered fluorite structure is adopted for A = Dy-Lu and Y (Doi et al., 2009). This upper limit of the parameter *ρ*_*a*_, (*i.e.*, on the relative size of cations with respect to the oxygen), seems to apply even to disordered fluorite oxides for which the cation:oxygen ratio is not 4:7. For example, *A*_2_TiO_5_ oxides form a disordered fluorite structure if A = Er-Lu (Aughterson et al., 2018a); together with studies on multiple *A*_x_Yb_1-x_TiO_5_ solid solution series by Aughterson et al. (2014), Aughterson et al. (2018a), and Aughterson et al. (2018b), this corresponds to a critical *ρ*_*a*_ value of 0.755–0.756, in excellent agreement with previous examples for oxides *A*_2_Zr_2_O_7_ and *A*_3_NbO_7_ with different stoichiometries. Though the *A*_3_TaO_7_ family of oxides form disordered fluorite structures for *A* = Ho-Lu, a reliable value for Pauling’s empirical crystal radius does not exist in literature; thus, no calculations on tantalate oxides were performed for this study.

**FIGURE 5:**
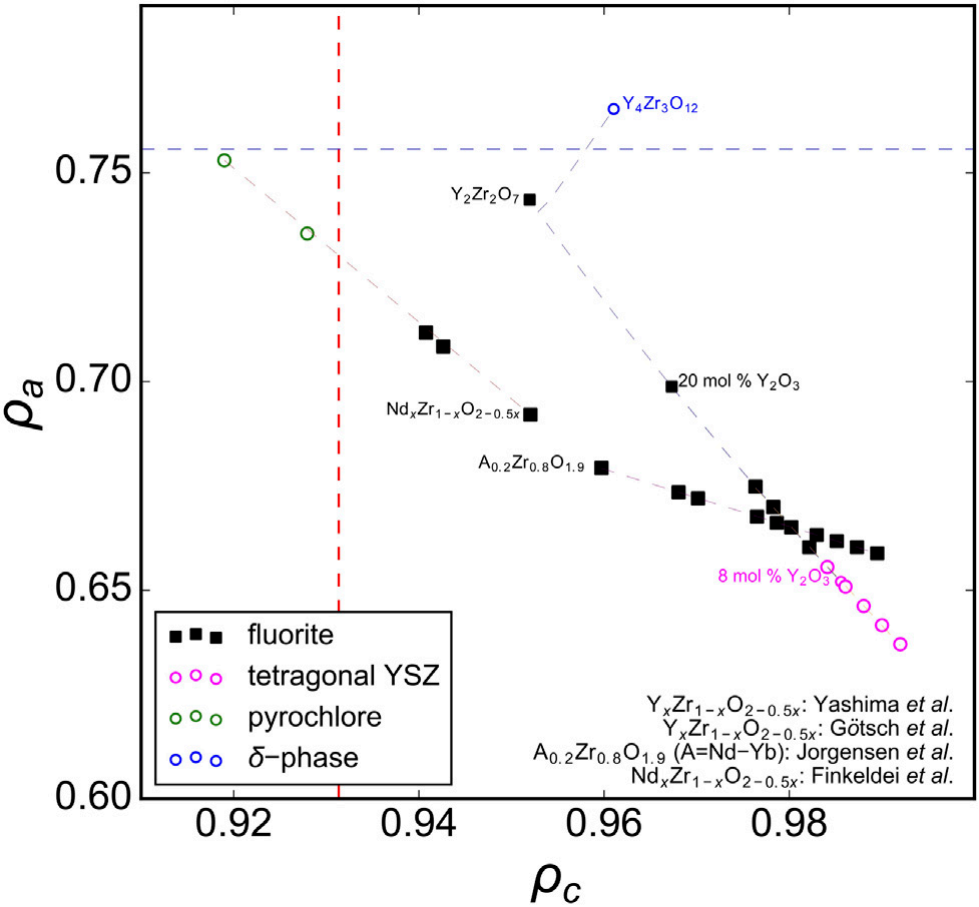
The phase space of A_x_Zr_1-x_O_2-0.5x_ (stabilized zirconia with A = Ln and Y) oxides spanned by parameters *ρ*_*a*_ and *ρ*_*c*_. Experimental data are taken from Jorgensen and Rittersh (1967), Yashima et al. (1993), Götsch et al. (2016), and Finkeldei et al. (2017) with reported structures that correspond to disordered fluorite (solid black squares), tetragonal YSZ (pink open circles), ordered pyrochlore (open green circles), and the delta phase (open blue circles). The dashed red vertical and blue horizontal lines represent refined *ρ*_*c*_ and *ρ*_*a*_ boundaries, respectively as determined in Figures 2–4.

Now that the upper *ρ*_a_ limit for the disordered fluorite stability field is determined, the lower limit must be found, which can be accomplished by stabilized-zirconia (*A*_*x*_Zr_1-*x*_O_2-0.5*x*_) compounds (Figure 5). Experimental data with different dopant levels of *A* = Y (Yashima et al., 1993; Götsch et al., 2016), *A* = Nd (Finkeldei et al., 2017), or different dopants at the same weight fractions (Jorgensen and Rittersh, 1967) suggest that the phase boundary between disordered fluorite and lower-symmetry ZrO_2_-type structures is *ρ*_*a*_ ∼ 0.657. This is just above the data point for 8 mol% yttria-stabilized zirconia (YSZ), which is generally accepted to be of a tetragonal form at room temperature (Götsch et al., 2016). Higher doping levels in YSZ produce disordered fluorite and doping beyond the upper *ρ*_*a*_ limit yields the delta-phase (Y_4_Zr_3_O_12_). Neodymia-stabilized zirconia (NSZ) also follows this behavior and lies within the disordered fluorite stability field; however, samples with higher Nd doping levels form an ordered pyrochlore structure (Nd_2_Zr_2_O_7_) if the *ρ*_*c*_ of the composition is smaller than the boundary established with ternary hafnate oxides (*A*_2_Hf_2_O_7_) above.

**FIGURE 6:**
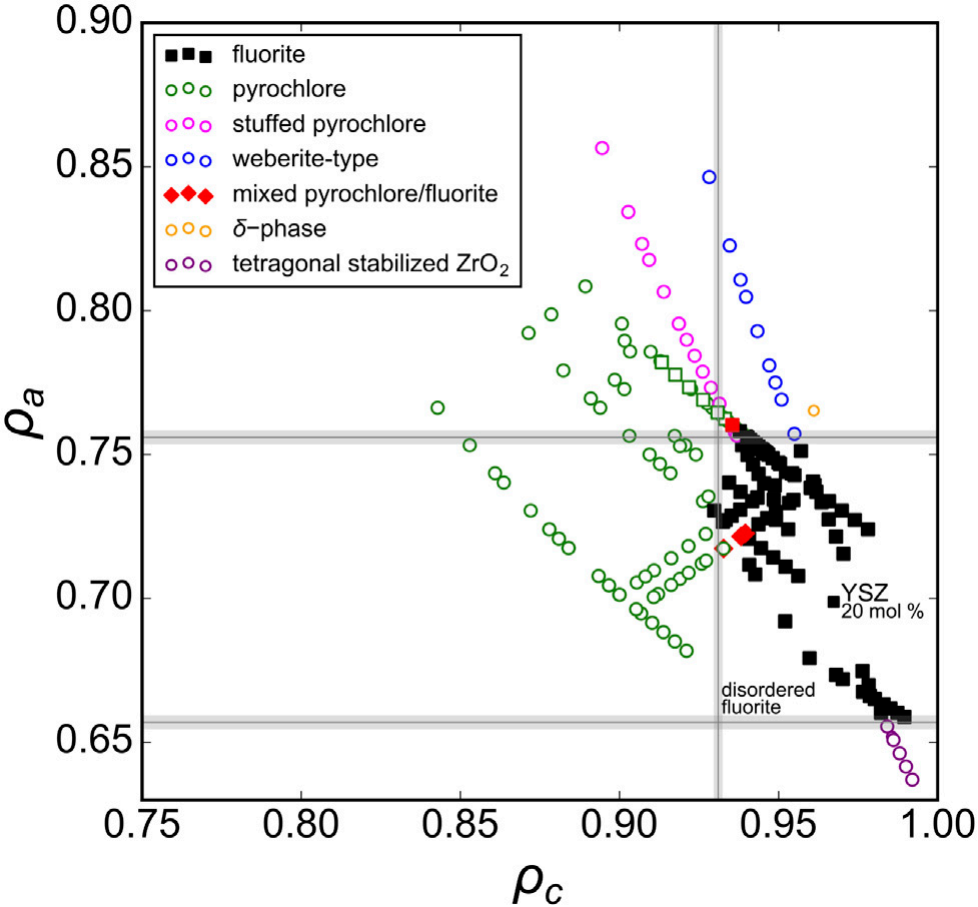
Experimental data with a wide range of reported structures for A_2_B_2_O_7_, A_2_BO_5_, A_3_BO_7_, and A_*x*_B_1-*x*_O_2-0.5*x*_ oxides represented in the phase space created by the parameters ρ_a_ and ρ_c_ (defined by Eqs 2, 3). The different symbols are defined in the legend and the gray lines denote the univalent radii boundaries that define the stability field of the disordered fluorite structure.

When all experimental data previously discussed are presented together in one phase diagram (Figure 6), a distinct region of disordered fluorite is apparently constrained by critical values of *ρ*_*c*_ and *ρ*_*a*_. The “left” boundary, (*ρ*_*c*_)_min_ = 0.931(1), can be interpreted as Goldschmidt’s first rule for ionic mixing (the uncertainty arises from considering the phase boundaries identified by multiple studies). Compounds that have *ρ*_*c*_ < (*ρ*_*c*_)_min_ consist of cations that are too dissimilar in their size to occupy the fluorite structure’s single cubic coordination polyhedron. The “upper” and “lower” boundaries, 0.657(2) ≤ *ρ*_*a*_ ≤ 0.756(2), are related to Pauling’s first rule which defines stable polyhedral configurations for cations based on their relative size to the surrounding anions. The lower boundary, (*ρ*_*a*_)_min_ = 0.657(2), can be interpreted as an application of Pauling’s first rule (“no rattle rule”) for the lower limit of 8-fold coordination. For disordered fluorite, the average anion radius accounts for intrinsic stoichiometric vacancies which corresponds to a total cation coordination with eight oxygen positions (*e.g.,* cation coordination with seven oxygen and one vacancy is equivalent to cation coordination with eight anions with radius 7/8 *r*_*O*_). Similarly, the upper boundary, (*ρ*_*a*_)_max_ = 0.756(2), coincides with the upper limit of cation coordination with seven oxygen and one vacancy. For a larger cation size this coordination configuration can no longer be maintained and ordering of the anion sublattice occur away from disordered fluorite. In summary, the disordered fluorite structure forms for oxides when the following two conditions are simultaneously satisfied:

$$i=\;\rho_{c}>\;0.931\left(1\right)\;and\;0.657\left(2\right)<\rho_{a}<\;0.756\left(2\right)$$ (4)

**FIGURE 7:**
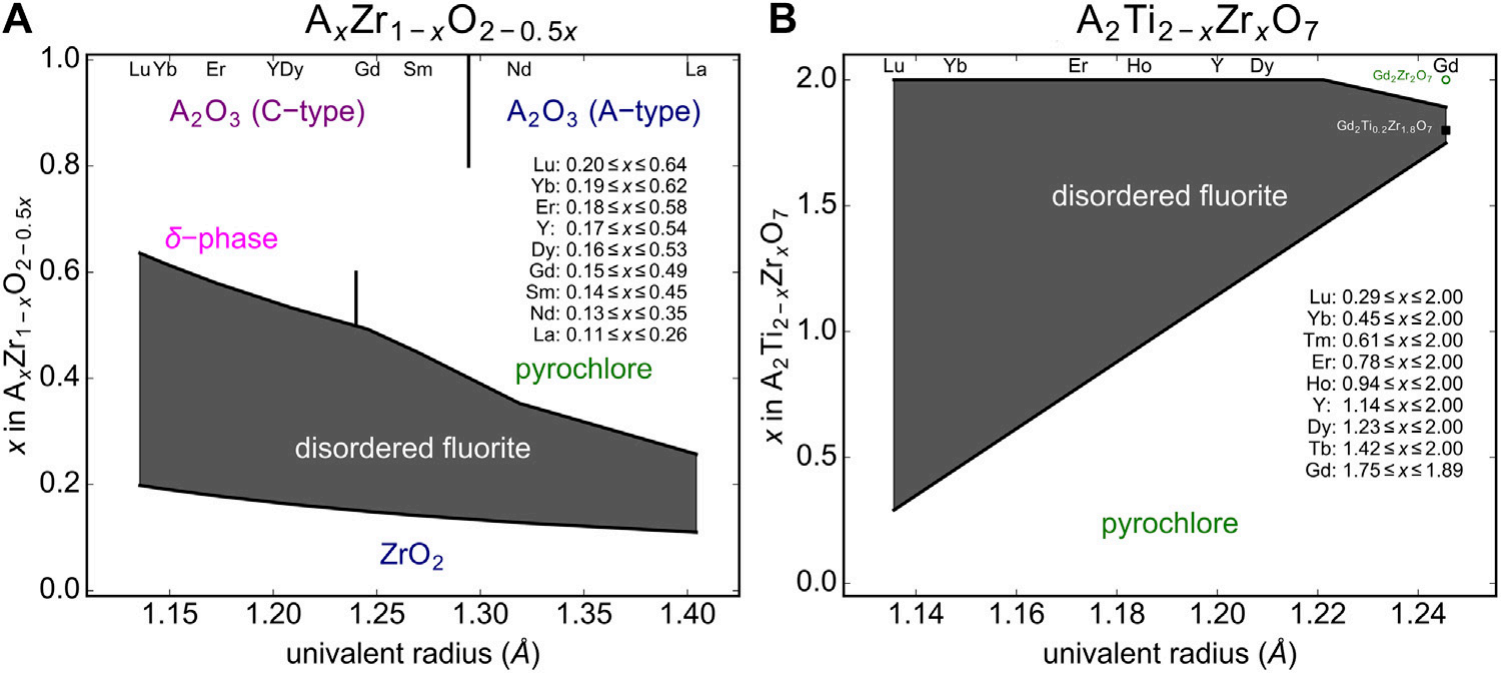
Predicted disordered fluorite stability field (gray shaded region) for **(A)** stabilized zirconia compounds (A_*x*_Zr_1-*x*_O_2-0.5*x*_) and **(B)** A_2_Ti_2-*x*_Zr_*x*_O_7_ solid-solution series with A representing the variable cation. The phase boundaries are established by the compositional parameter *x* and the univalent radius limits based on relations given in Eq. 4. Neighboring phases are shown for zirconia (ZrO_2_), sesquioxides (cubic “C-type” and trigonal “A-type” A_2_O_3_), δ-phases (A_4_Zr_3_O_12_), and pyrochlores (A_2_B_2_O_7_).

Using this structural stability field, we predict the compositional range over which disordered fluorite will form for stabilized zirconia compounds (Figure 7A). It is well known that for low values of *x* in *A*_*x*_Zr_1-*x*_O_2-0.5*x*_, a monoclinic or tetragonal ZrO_2_ structure will form (Clausen and Hayes, 1999; Navrotsky, 2010; Götsch et al., 2016). Cubic, disordered fluorite forms above a critical *x* value, which is determined by the size of the *A*-cation determined; the larger the *A*-cation, the lower the *x*. Based on relation (Sickafus et al., 2005) we predict the critical compositions (*i.e.,* the minimum doping level *x*) that results in a disordered fluorite structure across the lanthanide series, and for the largest and smallest A-site cation we predict La_0.11_Zr_0.89_O_1.945_ and Lu_0.19_Zr_0.81_O_1.905_, respectively (Figure 7A). The compounds with intermediate sized *A*-site cations exhibit a minimum doping level, *x*, with a nearly linear relationship with respect to their univalent radius. There is also an upper limit for *x* above which disordered fluorite is no longer stable and we used relation (Sickafus et al., 2005) to predict the critical compositions (i.e., the maximum doping level *x*), and for the largest and smallest *A*-site cation we predict La_0.26_Zr_0.74_O_1.87_ and Lu_0.64_Zr_0.38_O_1.68_, respectively (Figure 7A). For higher doping levels across the Ln series several other phases form (e.g., pyrochlore for larger cations and -phase for smaller cations) that depend on the *A*-site cation size. The larger the *A*-site cation, the lower the critical *x*-value and the dependence on the univalent radius across the Ln series is steeper than for the lower phase boundary. This means that the phase region of disordered fluorite is wider in terms of doping level *x* for smaller *A*-site cations (e.g., Lu) as compared with larger *A*-site cations (e.g., La). The minimum predicted *x*-value for disordered fluorite stability is in a linear relation with the univalent radius; this is because all stabilized zirconia compositional series “cross” the lower boundary, (*ρ*_*a*_)_min_ = 0.657(2) with only the size of the *A*-cation dopant dictating how far “horizontally” the series travels before crossing (*ρ*_*a*_)_min_. The maximum predicted *x*-value, however, is determined either by the left boundary, (*ρ*_*c*_)_min_ = 0.931(1), or the upper boundary, (*ρ*_*a*_)_max_ = 0.756(2), depending on the A-cation; this effect is observed in Figure 5 and explains why the maximum predicted *x*-values are not in a strictly linear relation with the univalent radius. These predictions for the compositional range over which the disordered fluorite structure is stable are largely in agreement with previous studies (Thornber et al., 1970; Bukaemskiy et al., 2021).

The second example used to predict the stability field of disordered fluorite based on relation (Sickafus et al., 2005) and the univalent radii are A_2_Ti_2-*x*_ Zr_*x*_O_7_ solid-solution series across all lanthanide *A*-site cations (Figure 7B). Given the different stoichiometry, the stability field of disordered fluorite spanned by the minimum and maximum *x* value across all univalent radii has a different shape as compared to stabilized zirconia. However, the disordered fluorite structure is predicted to be stable for larger *x-*ranges for cations with smaller univalent radii (*e.g.,* Yb) as it was the case for stabilized zirconia. This is not surprising as the smaller lanthanides are more similar in size to Zr (and Ti), and thus will have larger *ρ*_*c*_ values. Our model predicts, for example, that while neither Gd_2_Ti_2_O_7_ nor Gd_2_Zr_2_O_7_ form disordered fluorites at ambient conditions, the intermediate compositions Gd_2_Ti_2-x_Zr_x_O_7_ fall within the disordered fluorite structural stability field for 1.75 ≤ *x* ≤ 1.89. Future experimental work should focus on this series to confirm these predictions.

## Conclusion

A simple set of rules is proposed to define the structural stability field of disordered fluorite. Using Pauling’s univalent radii, the radii associated for application in his first, “no-rattle” rule, two parameters were defined that create a two-dimensional phase space over which a range of complex oxides were evaluated. Comparison with experimental data showed that the one parameter, *ρ*_*c*_, which quantifies the relative size among cations, must be above 0.931(1) for a compound to adopt the disordered fluorite structure. This is interpreted as a straightforward application of Goldschmidt’s first rule for ionic mixing (ions may freely replace one another in crystals if their radii differ by less than 15%). The other parameter, *ρ*_*a*_, which quantifies the relative size of the cations to the anions, must lie between 0.657(2) and 0.756(2) for a compound to adopt the disordered fluorite structure. These boundaries are correlated with the minimum and maximum limit of structural stability for cation coordination with eight oxygen positions, using an oxygen radius accounting for stoichiometric vacancies; these rules are therefore a generalization of Pauling’s first “no-rattle” rule. These results offer a simple, but effective, way of determining whether a complex oxide may adopt the disordered fluorite structure and, thus, provides guidance in future synthesis endeavors for this important class of materials. The current approach considers only room temperature, equilibrium phases and does not consider far-from-equilibrium processing and synthesis which may expand the stability field of disordered fluorite.

## Data Availability

The raw data supporting the conclusions of this article will be made available by the authors, without undue reservation.
